# Multivariate Statistical Analysis for the Detection of Air Pollution Episodes in Chemical Industry Parks

**DOI:** 10.3390/ijerph19127201

**Published:** 2022-06-12

**Authors:** Xiangyu Zhao, Kuang Cheng, Wang Zhou, Yi Cao, Shuang-Hua Yang

**Affiliations:** 1 College of Chemical and Biological Engineering, Zhejiang University, Hangzhou 310027, China; zjuzxy@zju.edu.cn (X.Z.); kuangcheng@zju.edu.cn (K.C.); zhouwang@zju.edu.cn (W.Z.); caoyi2018@zju.edu.cn (Y.C.); 2 Institute of Zhejiang University-Quzhou, Quzhou 324000, China

**Keywords:** air pollution, excessive emissions, principal component analysis, Hotelling’s *T*^2^, squared prediction error Q, detectability

## Abstract

Air pollution episodes (APEs) caused by excessive emissions from chemical industry parks (CIPs) have resulted in severe environmental damage in recent years. Therefore, it is of great importance to detect APEs timely and effectively using contaminant measurements from the air quality monitoring network (AQMN) in the CIP. Traditionally, APE can be detected by determining whether the contaminant concentration at any ambient monitoring station exceeds the national environmental standard. However, the environmental standards used are unified in various ambient monitoring stations, which ignores the source–receptor relationship in the CIP and challenges the effective detection of excessive emissions in some scenarios. In this paper, an approach based on a multivariate statistical analysis (MSA) method is proposed to detect the APEs caused by excessive emissions from CIPs. Using principal component analysis (PCA), the spatial relationships hidden among the historical environmental monitoring data are extracted, and the high-dimensional data are projected into only two subspaces. Then, two monitoring indices, $T^{2}$ and *Q*, which represent the variability in these subspaces, are utilized to monitor the pollution status and detect the potential APEs in the CIP. In addition, the concept of APE detectability is also defined, and the condition for APE detectability is derived, which explains when the APEs can be detectable. A simulated case for a CIP in Zhejiang province of China is studied to evaluate the performance of this approach. The study indicates that the method can have an almost 100% APE detection rate. The real-world measurements of Total Volatile Organic Compounds (TVOC) at a 10-min time interval from 3 December 2020∼12 December 2020 are also analyzed, and 64 APEs caused by excessive TVOC emissions are detected in a total of 1440 time points.

## 1. Introduction

In recent years, the rapid development of chemical industry parks (CIPs) has raised severe environmental concerns [1]. Air pollution episodes (APE) caused by excessive emissions of atmospheric pollutants in CIPs have led to a serious air quality deterioration to the surrounding environment [2,3]. Long-term exposure to air pollutants, such as fine particulate matter (PM${}_{2.5}$), sulfur dioxide (SO${}_{2}$), and volatile organic compounds (VOCs), has been associated with increased negative health effects [4,5,6]. Living in proximity to emission sources has a greater risk of acute respiratory infections, asthma, lung cancer, and other respiratory diseases [7,8]. Therefore, it is an essential task of local authorities to monitor the air pollution caused by industrial activities [9,10,11,12]. For the purposes of air pollution monitoring, air quality monitoring networks (AQMN) consisting of multiple fixed monitoring stations have been set up in CIPs [10,11,12,13] to serve as data sources.

Normally, air pollution monitoring in CIPs includes two tasks: APE detection and source term estimation (STE) [14]. APE is defined as the pollution episode caused by excessive emissions from plants in CIPs, and the purpose of APE detection is to determine whether there are excessive emissions. Many scholars are devoted to research on STE problems, specifically, estimating the source-emission rates and locations [15,16,17,18] in CIPs. However, the problems associated with APE detection in CIPs have not attracted sufficient attention. As the prerequisite for subsequent STE, detecting APEs in a timely and accurate fashion will help the regulatory authorities make decisions about when to trace the pollution source, especially the ones who adopt STE methods based on unmanned aerial vehicles (UAV) in CIPs. Recently, benefited by its flexibility in measurement locations, UAVs with air quality monitoring sensors have been widely used in STE problems [19,20,21], which could provides measurements at various locations to estimate the emission rates and locations more accurately compared with the fixed AQMN. However, the flying time of UAVs is limited by the capacity of battery power [22], which could rarely support the continuous measurement of atmospheric contaminants such as the static AQMN. Hence, before estimating the pollution source terms, it is necessary to detect the APEs in order to determine the optimal timing to release the UAVs, which could improve the monitoring efficiency and avoid ineffective flights.

The widely adopted approach of detecting air pollution episodes in current practice is to determine whether the contaminant concentration at any monitoring point exceeds the national environmental ambient standard promulgated by the Environmental Protection Regulatory Agency. Ref. [23] reported the monitoring results of methyl mercaptan in a CIP in Shanghai by the AQMN with five ambient monitoring stations. There were 414 methyl mercaptan violation records identified by comparing the concentration of any monitoring station with the national standard. Using the same monitoring method, ref. [24] reported 142 exceeding records of particulate matter in an Indian mining area through the AQMN in a year.

However, the commonly adopted approach has some defects. Firstly, these methods hardly guarantee that all excessive emission incidents are detectable. This is because the relationship between the emissions and the ambient sensors is not taken into account in the common methods. The AQMN’s concentration measurements are determined not only by the plant emission rates, but also by the relative positions between sensors and emission sources and by meteorological situations. In fact, it is possible that no violations are identified when comparing measurements with ambient standards even though there are excess emissions. For example, when the monitoring station is far away from the emission source, the concentration measurement at the monitoring station may not exceed the ambient standard. Moreover, although hundreds of contaminants have been monitored by government agencies in many countries, the ambient standards of some pollutants have not yet been clarified, especially for some species of volatile organic compounds (VOCs) [25,26]. Therefore, due to the lack of ambient standards, it is hard to determine whether the APE occurs even when some contaminants can be detected.

To overcome the shortcomings described above, in this paper, a data-driven approach based on a multivariate statistical analysis (MSA) method, principal component analysis (PCA) specifically, is proposed to detect the APE caused by excessive emissions. PCA is the most common MSA method, and it aims to analyze the joint behavior and the inner relationship between high-dimensional variables [27]. It has been widely used in data dimension reduction [28], pattern recognition [29], image compression [30], and other various fields [31]. In the field of environmental protection, PCA is applied in some areas, such as source apportionment [32,33,34], evaluating the performance of AQMN and identifying redundant stations [35,36,37]. In addition, PCA is a powerful technique to detect abnormal behaviors in a process or system with correlated variables [38,39].

In CIPs, the concentration data from each sensor are affected by multiple emission sources at the park, and there is a linear correlation between the concentration measurements of each sensor [40,41]. In this study, a PCA model was employed to extract the inter-dependence and inherent statistical regularity between these concentration measurements. Using PCA, high-dimensional pollutant concentration measurements are partitioned into only two subspaces: a principal component subspace (PCS) and a residual subspace (RS). Then, two indices, Hotelling’s $T^{2}$ and the squared prediction error *Q*, are used to represent the variability in PCS and RS, respectively. The normal regions of these indices are determined by their statistical distribution based on historical measurements under normal emissions. Finally, an APE will be detected if the monitoring indices corresponding to the real-time data exceed the normal range.

Compared to the common method, the advantages of this statistical detection approach are as follows. Firstly, the pollution status of the whole park is monitored by only two indices ($T^{2}$ or *Q*) rather than high-dimensional concentration data in each station, which improves the efficiency of APE detection. Secondly, the thresholds of monitoring indices are determined specifically through historical measurements of CIPs rather than a unified environmental standard. Therefore, these statistical limits can delineate the normal range of air pollution better for a particular CIP. Thirdly, this method makes it possible to detect the APEs caused by pollutants that lack environmental standards, such as some VOC species.

The main contributions in this paper are as follows:

1. A statistical framework for APE detection based on the MSA method is proposed in this study. Under this framework, the pollution situation of the CIP is monitored as a whole rather than individually in each station.
2. The pollution status of the entire park is monitored using two indices, $T^{2}$ and *Q*, whose thresholds are determined by the statistical distribution of the indices based on historical measurements. By analyzing the source–receptor relationship in the framework of PCA, it has been proved in theory that these two indices can reveal changes in emission rates in most cases, implying that they are effective in detecting APEs due to excessive emissions. The simulation test also demonstrates the method’s high APE detection rate.
3. The concept of APE detectability is introduced, and the condition for APE detectability is also derived, which explains when the APEs can be detectable.

The rest of this paper is organized as follows. The relationship between the emission sources and the ambient receptors is explained in Section 2. Section 3 proposes the methodology of PCA for APE detection. Section 4 presents a simulation test to verify the feasibility of this method. The real-world application is demonstrated in Section 5. Section 6 concludes the study with the contributions, limitations, and further research opportunities.

## 2. Source–Receptor Relationship for CIPs

Atmosphere dispersion models are often adopted to describe the relationship between the source and receptor. Among these models, the Gaussian plume model [42] is widely used for non-reactive pollutants. Assume that the wind direction is along the *x*-axis, the *z*-axis is vertical, the source is at the origin, the receptor is at (*x*, *y*, *z*), and the concentration *c* measured in the receptor can be calculated based on the Gaussian Plume model:

(1) $$c=\frac{q}{2\pi uD_{y}D_{z}}\exp\left[-\frac{y^{2}}{2D_{y}^{2}}\right]\left\{\exp{\left[-\frac{{\left(z-h\right)}^{2}}{2D_{z}^{2}}\right]}^{2}+\exp{\left[-\frac{{\left(z+h\right)}^{2}}{2D_{z}^{2}}\right]}^{2}\right\}$$

where *q* is the emission rate of a point source at the origin, *h* is the height of the source in *z* direction, *u* is the wind speed. $D_{y(z)}$ are standard deviations of concentration along *y*-(*z*-)axis. Equation (1) can be simplified as follows:

(2) c=aq

where the coefficient *a* can be calculated as follows:

(3) $$a=\frac{1}{2\pi uD_{y}D_{z}}\exp\left[-\frac{y^{2}}{2D_{y}^{2}}\right]\left\{\exp{\left[-\frac{{\left(z-h\right)}^{2}}{2D_{z}^{2}}\right]}^{2}+\exp{\left[-\frac{{\left(z+h\right)}^{2}}{2D_{z}^{2}}\right]}^{2}\right\}$$

The coefficient *a* represents the concentration response in unit emission rate, which is determined by the wind direction, wind speed, and positions of the emission source and the ambient sensor. Equation (2) indicates that for a given wind direction and wind speed, the relationship between a receptor and an emission source is linear.

Usually, there are multiple receptors and multiple emission sources in a CIP. In non-reactant situations, the concentration at a receptor is the sum of concentrations produced by multiple sources. At time $t_{i}$, the relationship between the concentration measurements at *m* ambient monitoring points and the emission rates of *n* pollution sources can be represented as follows:

(4) $$\left\{\begin{array}{cc} c_{t_{i},1} & =a_{t_{i},1,1}q_{t_{i},1}+a_{t_{i},1,2}q_{t_{i},2}+\cdots +a_{t_{i},1,n}q_{t_{i},n}\\ c_{t_{i},2} & =a_{t_{i},2,1}q_{t_{i},1}+a_{t_{i},2,2}q_{t_{i},2}+\cdots +a_{t_{i},2,n}q_{t_{i},n}\\ c_{t_{i},3} & =a_{t_{i},3,1}q_{t_{i},1}+a_{t_{i},3,2}q_{t_{i},2}+\cdots +a_{t_{i},3,n}q_{t_{i},n}\\ \; & \cdots \\ c_{t_{i},m} & =a_{t_{i},m,1}q_{t_{i},1}+a_{t_{i},m,2}q_{t_{i},2}+\cdots +a_{t_{i},m,n}q_{t_{i},n} \end{array}\right.$$

where $c_{t_{i},w}$ is the measured concentration from the ambient monitoring point *w* at time point $t_{i}$, $q_{t_{i},j}$ is the emission rate of pollution source *j* at time $t_{i}$, and $a_{t_{i},w,j}$ is the coefficient between the emission source *j* and the monitoring point *w* at time $t_{i}$, for w=1,2,⋯,m and j=1,2,⋯,n. The matrix expression of Equation (4) is as follows:

(5) $$c_{t_{i}}=q_{t_{i}}A_{t_{i}}^{{}^{\prime }}$$

where $c_{t_{i}}=\left[c_{t_{i},1},c_{t_{i},2},\cdots ,c_{t_{i},m}\right]\in {ℜ}^{1\times m}$, $q_{t_{i}}=\left[q_{t_{i},1},q_{t_{i},2},\cdots ,q_{t_{i},n}\right]\in {ℜ}^{1\times n}$, $A_{t_{i}}=[a_{t_{i},1},a_{t_{i},2},\cdots ,a_{t_{i},n}]\in {ℜ}^{m\times n}$ is the coefficient matrix, $a_{t_{i},j}={\left[a_{t_{i},1,j},a_{t_{i},2,j},\cdots ,a_{t_{i},m,j}\right]}^{{}^{\prime }}$, j=1,2,⋯,n, and $A_{t_{i}}^{{}^{\prime }}$ denotes the transpose of matrix $A_{t_{i}}$.

Assume the wind direction and wind speed are fixed during *k* time points, $t_{1}$, $t_{2}$, ⋯, $t_{k}$. Therefore, the coefficient matrices at various time points, $A_{t_{1}}$, $A_{t_{2}}$, ⋯, $A_{t_{k}}$, are the same, which can be expressed by a fixed matrix $A=\left[a_{1},a_{2},\cdots ,a_{n}\right]\in {ℜ}^{m\times n}$, where $a_{j}={\left[a_{1,j},a_{2,j},\cdots ,a_{m,j}\right]}^{{}^{\prime }}$, j=1,2,⋯,n. Correspondingly, the historical concentration data are $C={\left[c_{t_{1}}^{{}^{\prime }},c_{t_{2}}^{{}^{\prime }},\cdots ,c_{t_{k}}^{{}^{\prime }}\right]}^{{}^{\prime }}\in {ℜ}^{k\times m}$ and the matrix of emissions rates is $Q={\left[q_{t_{1}}^{{}^{\prime }},q_{t_{2}}^{{}^{\prime }},\cdots ,q_{t_{k}}^{{}^{\prime }}\right]}^{{}^{\prime }}\in {ℜ}^{k\times n}$. Equation (5) can be extended as follows:

(6) $$C={\mathrm{QA}}^{{}^{\prime }}$$

Usually, the plants in CIPs operate independently [43], which means that there is no necessary relationship between the emission rates of each plant. Therefore, the emission rates of each plant can be regarded as independent of the others and the covariance matrix B of the emission rates is diagonal. Assume that Q follows the multiple Gaussian distribution N(μ,B), where $\mu =[\mu_{q,1},\mu_{q,2},\cdots ,\mu_{q,n}]$, $B=diag(\sigma_{q,1}^{2},$ $\sigma_{q,2}^{2},\cdots ,\sigma_{q,n}^{2})$, $\mu_{q,i}$ and $\sigma_{q,i}$ are the mean and standard deviation of the emission rate at *i*th source, for i=1,2,⋯,n. According to Equation (6), C is the linear combination of Q. Hence, $C∼N({\mu A}^{{}^{\prime }},{\mathrm{ABA}}^{{}^{\prime }})$. In most circumstances, ${\mathrm{ABA}}^{{}^{\prime }}$ is not a diagonal matrix, which means that the concentration data of various monitoring points are related. For this reason, the correlations between the variables are ignored using common univariate methods to monitor air pollution and MSA approaches, which can reflect the inner correlation among the various variables and may be more suitable for detecting APEs in CIPs.

## 3. Methodology

### 3.1. Principal Component Analysis

PCA is the most common MSA method, which is an orthogonal linear transformation that transforms the data into a new coordinate system that maximizes the variance of the variables. Using PCA, the data can be projected into a lower-dimensional space in a way that can optimally preserve the correlation between the variables and extract the variability in the data [44]. By PCA, the data from multiple sensors of AQMN can be projected into only two subspaces, principal component subspace (PCS) and residual subspace (RS), respectively, which make the air pollution monitoring simpler and more effective.

The procedure of feature extraction through the PCA can be formulated as:

**Step** **1:** Normalize the initial data C to zero mean and unit variance as follows:
  (7) $$Z=\Delta C\sigma^{-1}$$
  where $Z\in {ℜ}^{k\times m}$ is the normalized data matrix representing the change in concentration measurements; $\sigma =diag(\sigma_{c,1},\sigma_{c,2},\cdots ,\sigma_{c,m})$, $\Delta C={\left[\Delta c_{t_{i},j}\right]}_{k\times m}$, $\Delta c_{t_{i},j}=c_{t_{i},j}-\mu_{c,j}$ for i=1,2,…,k and j=1,2,…,m; and $\mu_{c,j}$ and $\delta_{c,j}$ represent the mean and standard deviation of the concentration values measured at *j*th sensor, respectively. After this step, all the data are normalized in the same manner.
**Step** **2:** Perform eigenvalue decomposition on the covariance matrix:
  (8) $$D=\frac{1}{m-1}Z^{{}^{\prime }}Z$$
  (9) $$D=P\Lambda P^{{}^{\prime }}$$
  where the diagonal matrix $\Lambda \in {ℜ}^{m\times m}$ contains progressively decreasing non-negative eigenvalues ($\lambda_{1}\geq \lambda_{2}\geq \lambda_{3}\geq \cdots \geq \lambda_{m}\geq 0$). $P\in {ℜ}^{m\times m}$ is the loading matrix with $P^{{}^{\prime }}P=I$. The principal component transformation is given by
  (10) S=ZP
  where the S is the score matrix. Equivalently, Z is decomposed by PCA as:
  (11) $$Z={\mathrm{SP}}^{{}^{\prime }}$$
  After this step, the initial data matrix is decomposed into two matrices, the loading matrix P and the score matrix S. The loading matrix contains the coefficients of the linear combination of the initial variables from which the principal components are constructed, and the score matrix represents the principal components.
**Step** **3:** Determine the number of principal components *r* by calculating the Cumulative Percent Variance (CPV) *a*:
  (12) $$a=\frac{\sum_{i=1}^{r}\lambda_{i}}{\sum_{i=1}^{m}\lambda_{i}}$$
  The principal components are considered to retain predominant information of raw data when a≥95%. The loading matrix P and the score matrix S can be divided as follows:
  (13) $$P=[P_{r}\;{\hat{P}}_{r}]$$
  (14) $$S=[S_{r}\;{\hat{S}}_{r}]$$
  where $P_{r}$ and $S_{r}$ are the first *r* columns of P and S, respectively. As a result, the PCA model structure is illustrated as follows:
  (15) $$\begin{array}{cc} Z & ={\mathrm{SP}}^{{}^{\prime }}\\ \; & =\left[S_{r}\;{\hat{S}}_{r}\right]{\left[P_{r}\;{\hat{P}}_{r}\right]}^{{}^{\prime }}\\ \; & =S_{r}P_{r}^{{}^{\prime }}+{\hat{S}}_{r}{\hat{P}}_{r}^{{}^{\prime }}\\ \; & =\tilde{Z}+E \end{array}$$
  where $\tilde{Z}=S_{r}P_{r}^{{}^{\prime }}$ is the estimation of Z and $E={\hat{S}}_{r}{\hat{P}}_{r}^{{}^{\prime }}$ is the residual part of the PCA model, which will then be utilized to construct the indices of APE detection in PCS and RS, respectively.

### 3.2. The Indices of APE Detection

**Figure 1:**
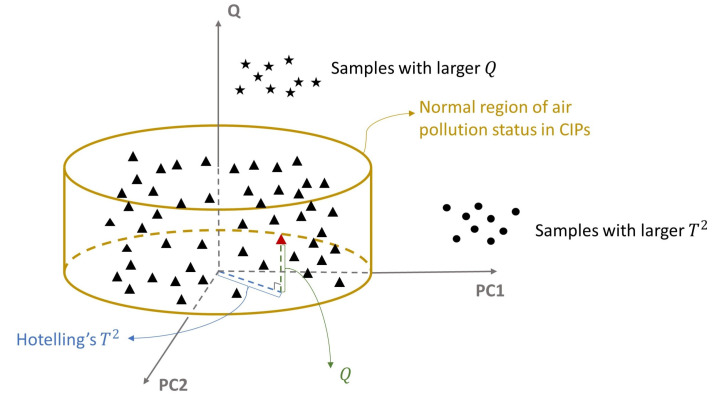
Graphical interpretation of $T^{2}$ and *Q* with two principal components (PC1 and PC2). All samples are projected into PCS and RS using PCA. The triangles represent the samples under normal emissions, and the cylinder defines the normal region of air pollution status in the CIP. The five-pointed stars denote the samples with larger *Q*, while the circles represent the samples with larger $T^{2}$.

After feature extraction using PCA model, the raw high-dimensional data are projected into two orthogonal subspace [38], principal component subspace (PCS), and residual subspace (RS), respectively. Then, Hotelling’s $T^{2}$ and squared prediction error (SPE), *Q*, are built to detect the APEs, which represent the variability in PCS and RS, respectively. A graphical interpretation of $T^{2}$ and *Q* with two principal components (PC1 and PC2) is illustrated in Figure 1.

The statistical $T^{2}$ and *Q* of time $t_{i}$ is calculated as follows:

(16) $$T^{2}=z_{t_{i}}P_{r}\Lambda_{r}^{-1}P_{r}^{{}^{\prime }}z_{t_{i}}^{{}^{\prime }}$$

(17) $$Q=\left(z_{t_{i}}-{\hat{z}}_{t_{i}}\right){\left(z_{t_{i}}-{\hat{z}}_{t_{i}}\right)}^{{}^{\prime }}=z_{t_{i}}\left(I_{m}-P_{r}P_{r}^{{}^{\prime }}\right){\left(I_{m}-P_{r}P_{r}^{{}^{\prime }}\right)}^{{}^{\prime }}z_{t_{i}}^{{}^{\prime }}$$

where $z_{t_{i}}$ is a 1×m normalized concentration vector at time $t_{i}$ and $\Lambda_{r}$ is leading principal minor of order *r* of matrix Λ.

It can be proved that the change in ambient concentration measurements $z_{t_{i}}$ can be reflected in $T^{2}$ or *Q*, which is illustrated mathematically in Lemma 1.

**Lemma** **1.**  *For the PCA, if $z_{t_{i}}\neq 0$, then, either $T^{2}\neq 0$ or Q≠0.*

**Proof of Lemma** **1.** Assume $z_{t_{i}}\neq 0$.

If

(18) $$T^{2}=z_{t_{i}}P_{r}\Lambda_{r}^{-1}P_{r}^{{}^{\prime }}z_{t_{i}}^{{}^{\prime }}=t_{t_{i}}\Lambda_{r}^{-1}t_{t_{i}}^{{}^{\prime }}=0,$$

where $t_{t_{i}}$ is a 1×r score vector in PCS, $t_{t_{i}}=0$ because of $rank(\Lambda_{r}^{-1})=r$. Thus,

(19) $$Q=∥z_{t_{i}}\left(I_{m}-P_{r}P_{r}^{{}^{\prime }}\right){∥}^{2}=∥\left(z_{t_{i}}I_{m}-z_{t_{i}}P_{r}P_{r}^{{}^{\prime }}\right){∥}^{2}=∥\left(z_{t_{i}}I_{m}-t_{t_{i}}P_{r}^{{}^{\prime }}\right){∥}^{2}={∥z_{t_{i}}I_{m}∥}^{2}\neq 0.$$

On the other hand, if

(20) Q=0,

there is no residual that means that E=0 and $\tilde{Z}=Z$, as shown by Equation (15). Therefore, the number of principal components r=m and

(21) $$T^{2}=z_{t_{i}}P\Lambda^{-1}P^{{}^{\prime }}z_{t_{i}}^{{}^{\prime }}\neq 0,$$

due to $rank(P\Lambda^{-1}P^{{}^{\prime }})=m$.

This proves that $T^{2}$ and *Q* cannot be 0 at the same time that $z_{t_{i}}\neq 0$. □

### 3.3. The APE Detectability

The APE detectability explains whether the APEs can be detected using the monitoring indices $T^{2}$ and *Q*. Lemma 1 represents the change in ambient measurements $z_{t_{i}}$ can result in $T^{2}$ and *Q*. Therefore, an APE can be detectable only when the emission rates $\Delta q_{t_{i}}$ are reflected in $z_{t_{i}}$. Here, the APE detectability is defined as follows.

**Definition** **1.**  *An APE is detectable by a set of normalized measurements, $z_{t_{i}}$, if any $\Delta q_{t_{i}}\neq 0$ will result in $z_{t_{i}}\neq 0$.*

Based on the above definition, the necessary and sufficient condition for an APE to be detectable is given in Theorem 1.

**Theorem** **1.**  *For an air pollution monitoring system in (5), an APE is detectable by $z_{t_{i}}$ if and only if*

(22) rank(A)=n

**Proof of Theorem** **1.** According to the system in (5) and Equation (7), $z_{t_{i}}$ is calculated as:

(23) $$z_{t_{i}}=\Delta c_{t_{i}}\sigma^{-1}=\Delta q_{t_{i}}A^{{}^{\prime }}\sigma^{-1}$$

where $\Delta q_{t_{i}}$ is the change in the emission rates from the mean at time $t_{i}$. An APE can be detectable as long as any $\Delta q_{t_{i}}\neq 0$ can result in $z_{t_{i}}\neq 0$. It is equivalent that $z_{t_{i}}$ has no non-zero solution in the equation:

(24) $$\Delta q_{t_{i}}A^{{}^{\prime }}\sigma^{-1}=0$$

whose necessary and sufficient condition is:

(25) $$rank\left(A^{{}^{\prime }}\sigma^{-1}\right)=rank\left(A^{{}^{\prime }}\right)=rank\left(A\right),$$

because $rank(\sigma^{-1})=m$. □

**Corollary** **1.**  *If an APE is detectable, then it is detectable by either $T^{2}$ or Q.*

**Proof of Corollary** **1.** The corollary can be derived by applying both Theorem 1 and Lemma 1. □

Theorem 1 demonstrates that whether an APE can be detectable depends on the rank of the coefficient matrix A, which is affected by the relative location of pollution sources and ambient sensors and real-time meteorological conditions. Only if the condition above is satisfied can the change of the emission rate be reflected in the indices $T^{2}$ and *Q* in any situation, and the PCA method for APE detection is feasible. If the condition is not met, the APE may be undetectable and the plume emitted from some sources may not be captured by all the static ambient monitoring sensors under certain weather conditions.

**Figure 2:**
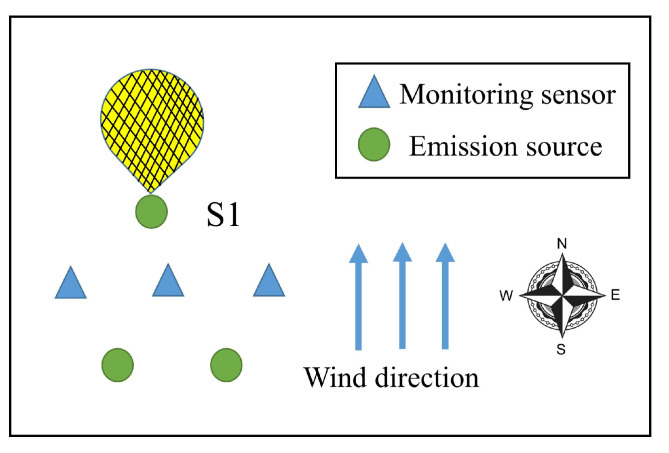
Undetectable case.

Figure 2 shows a simple undetectable case to illustrate the concept of APE detectability and its influencing factors. There are 3 monitoring stations and 3 emission sources, and the wind direction is northward. In this wind direction, rank(A)<n=3. The area marked by the grid represents the plume from the source S1, which cannot be detected by all the monitoring sensors. When S1 emits the contaminants excessively and the emission rates of other sources are assumed to be the same as the mean, the concentration measurements are unchanging no matter how large the emission rate of S1 is, and the indices $T^{2}$ and *Q* are zero, which means that the approach of the APE detection is not applicable in this situation. Actually, this condition is the same as the condition of traceability proposed in [22,40,41], which explains when the emission rates of the sources can be estimated uniquely using least squares. This illustrates whether an APE can be detectable, and whether the emission source can be traceable relies on totally identical criteria.

It is worth noting that MSA methods are also widely used in fault detection and diagnosis [45]. Many scholars have focused on improving the detecting methods to increase the fault detection accuracy. However, the detectability of the fault is ignored in most past studies. For a fault with low detection accuracy, this may be because the fault itself is not detectable, rather than the method not being good enough. If the condition of detectability is not satisfied, it will be hard to detect the fault because the measurements barely change in this situation.

### 3.4. Thresholds of the APE Detection Indices

The anomaly detector is trained by analyzing the distribution of $T^{2}$ and *Q* computed by the historical concentration data of *k* time points. Because the distribution of the concentration may not be Gaussian in the strict sense, the probability density functions (PDFs) of these two indices are estimated directly through Kernel Density Distribution (KDE). The upper control limits $T_{UCL}^{2}$ and $Q_{UCL}$ can be obtained by $P(T^{2}<T_{UCL}^{2})=\alpha$ and $P(Q<Q_{UCL})=\alpha$, with

(26) $$P\left(T^{2}<T_{UCL}^{2}\right)=\int_{-\infty }^{T_{UCL}^{2}}\frac{1}{qh}\sum_{k=1}^{q}K\left(\frac{T^{2}-T_{k}^{2}}{h}\right)dT^{2}$$

(27) $$P\left(Q<Q_{UCL}\right)=\int_{-\infty }^{Q_{UCL}}\frac{1}{qh}\sum_{k=1}^{q}K\left(\frac{Q-Q_{k}}{h}\right)d\left(Q\right)$$

where $T_{k}^{2}$ and $Q_{k}$ are samples of $T^{2}$ and *Q*, h is the bandwidth, and K(·) is the kernel function. The selected kernel function is:

(28) $$K\left(v\right)=\frac{1}{\sqrt{2\pi }}exp\left(-\frac{v^{2}}{2}\right)$$

More details for KDE are given in [46].

When the real-time concentration data from the AQMN arrive at a specific time, $T^{2}$ and *Q* are calculated through Equations (16) and (17) to detect an APE. The air pollution status is considered abnormal if the following condition is satisfied:

(29) $$T^{2}>T_{UCL}^{2}\;or\;Q>Q_{UCL}$$

### 3.5. Influence of the Meteorological Conditions

In the analysis above, the meteorological conditions, mainly including the wind speed and wind direction, are assumed to be unvarying. However, in the actual situation, the weather conditions are changing all the time. This means that the coefficient matrices in various time points are different, and Equation (5) cannot be extended as Equation (6), which has an impact on the PCA method proposed above. Therefore, some changes are needed to ensure that Equation (6) can be satisfied.

#### 3.5.1. Wind Speed

Wind speed is inversely proportional to the concentration data *c* in most instances. Hence, by multiplying the concentration by the wind speed, Equation (6) can be changed as follows:

(30) $$C_{0}=QA_{0}^{{}^{\prime }}$$

where $C_{0}={\left[u_{t_{1}}c_{t_{1}}^{{}^{\prime }},u_{t_{2}}c_{t_{2}}^{{}^{\prime }},\cdots ,u_{t_{k}}c_{t_{k}}^{{}^{\prime }}\right]}^{{}^{\prime }}\in {ℜ}^{k\times m}$ and $A_{0}^{{}^{\prime }}$ is the coefficient matrix in unit speed. In this way, the data in various time points can be merged.

#### 3.5.2. Wind Direction

From Equation (1), it can be seen that the influence of the wind direction on the concentration data is highly non-linear, and it is hard to separate the impact of wind direction through simple mathematical transformations. From Equation (23), $\Delta q_{t_{i}}$ can be reflected in $z_{t_{i}}$ if the condition of APE detectability proposed in Theorem 1 is satisfied. However, if the wind direction fluctuates, A changes simultaneously. Consequently, it is difficult to judge why the indices exceed the thresholds. The exceeding record may be due to violent fluctuations in wind direction or because of excessive emissions.

To deal with variant wind directions, it is assumed that wind directions varying within a certain small range are constant. Then, the whole historical receptor dataset can be classified into a number of subsets based on the wind direction records. In each subset, the corresponding wind directions are all within a relatively small range; hence, the receptor data of the same subset can be used for PCA.

### 3.6. The Whole Workflow of APE Detection Using PCA

**Figure 3:**
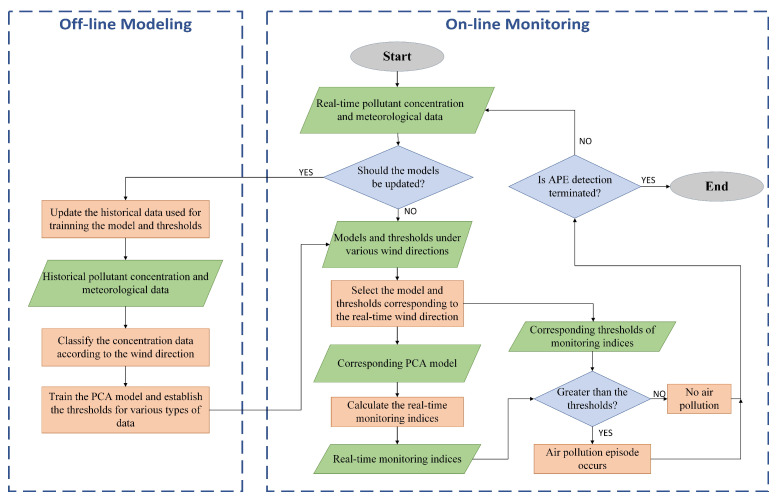
The overall workflow diagram of APE detection.

The overall workflow diagram is shown in Figure 3. The PCA approach for APE detection has two phases, offline modeling and on-ine monitoring. In the offline phase, the concentration data are divided into some subsets according to the wind direction in the first step. Then, the PCA models and monitoring thresholds of all the subsets are created. In the online phase, based on the direction, the corresponding thresholds and model are chosen first. After that, whether the APE occurred is determined based on the real-time measurements from AQMN.

## 4. Simulation Experiment

**Figure 4:**
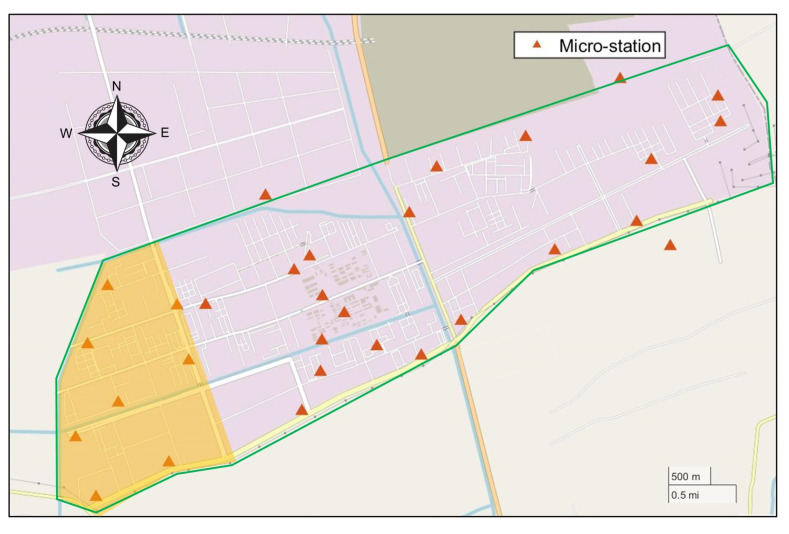
Map of the CIP. The green line represents the boundary of the CIP.

The studied case is a chemical industry park (shown in Figure 4) about 21 km${}^{2}$ in Zhejiang province, China, and some chemical enterprises specializing in dyes, pharmaceuticals and so on are located there. More than 100 air pollution emission sources have a serious impact on the local environment. Therefore, in the CIP, 30 ambient micro-stations were set up to monitor the concentration of contaminants (PM${}_{2.5}$, PM${}_{10}$, SO${}_{2}$, NO${}_{X}$, TVOC, etc.) and meteorological conditions (wind direction, wind speed, etc.).

To validate the approach proposed above, the concentration data in normal and abnormal emission situations are required. However, it is difficult to judge whether the actual concentration data are in a normal or abnormal emission situation. Therefore, a simulation test is conducted initially.

**Figure 5:**
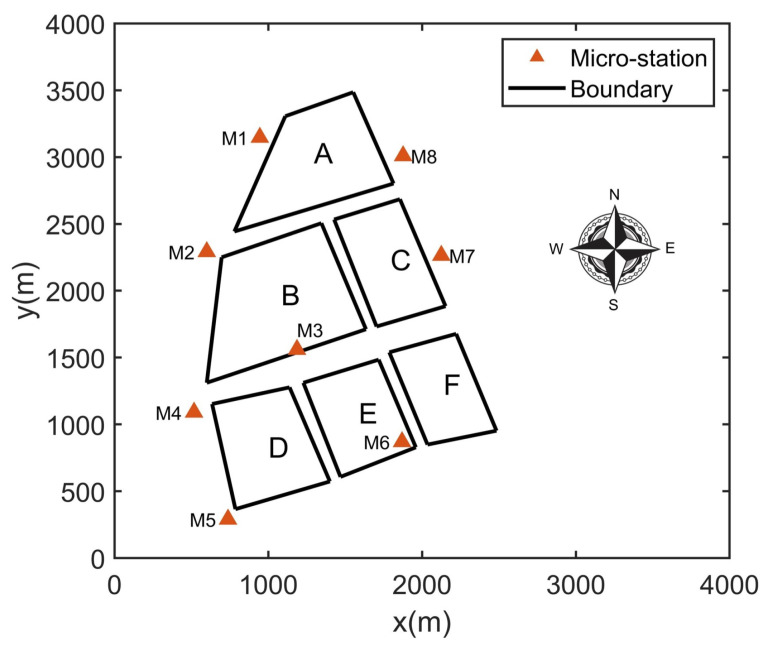
Location of monitoring stations and pollution sources.

In order to make the case easier to describe and verify the feasibility of the method above more effectively, the scene is simplified in the simulation experiment. A part of the zone in the west of a CIP (the yellow shaded area in Figure 4) is selected. In this zone, there are six plants (A, B, C, D, E, and F) and eight fixed ambient micro-stations ($M_{1}∼M_{8}$) fitted in the zone. The location diagram is shown in Figure 5. There are two assumptions in this experiment:

1. This zone is regarded as independent in the simulation experiment, which means that the concentration monitored in these eight micro-stations are triggered only by the six plants in the zone and not affected by the plants in other zones of the CIP;
2. Each plant is equivalent to one emission source and only one non-reactive contaminant is considered.

### 4.1. Data Simulation

To generate enough scenarios, the variation in two kinds of parameters, (1) emission rate and (2) wind direction (mainly affecting the coefficients in Equation (4), should be taken into consideration. The wind speed is set to 1 m/s in the simulations because all scenarios in various speeds can be made equivalent to those in unit wind speed by multiplying the concentration by the wind speed. The coefficient $a_{t,i,j}$ is calculated through the American Meteorological Society and Environmental Policy Agency Regulatory Model (AERMOD) developed by the U.S Environmental Protection Agency (EPA) [47]. It is a Gaussian dispersion based model, and the localization parameter settings in this model are shown in Table 1.

Training data and test data are simulated.

#### 4.1.1. Training Data

One thousand groups of concentration data within the normal range of emission rates are simulated to train the PCA model and establish the thresholds of APE detection indices. It is assumed that the emission rates fluctuate within a certain range of the mean under normal circumstances, which follow the Gaussian distribution. The specific source rates are shown in Table 2.

For the wind directions, take $130^{\circ }∼140^{\circ }$, which is regarded as a type, as an example for analysis. The wind direction data simulated follows uniform distribution U(130,140). Finally, the concentration data are calculated through AMEROD. White Gaussian noises are added into the simulated data, which follows the Gaussian distribution N(0,0.05).

#### 4.1.2. Test Data

To test the performance of the APE detection method, two groups of test data are simulated. Firstly, the data in the abnormal situation with excessive emissions are generated to test the APE detection rate. It is assumed that the APE can only be caused by one plant emitting excessively. Therefore, six kinds of APE caused by excess emissions are studied. Descriptions of the APEs studied are presented in Table 3. Here, the excessive emission, specifically, is defined as the mean emission rate being 2 times the mean in the context of normal emission. Wind directions are set at $135^{\circ }$ in test data. One thousand groups of concentration data caused by each APE are generated in the same way as the training data.

Secondly, because an APE may also be detected under normal emission rates, 1000 sets of concentration data in normal emissions are also simulated to evaluate the false alarming rate of this method. The wind directions are also set at $135^{\circ }$.

### 4.2. Results of Simulation Test

#### 4.2.1. PCA Model Construction

**Figure 6:**
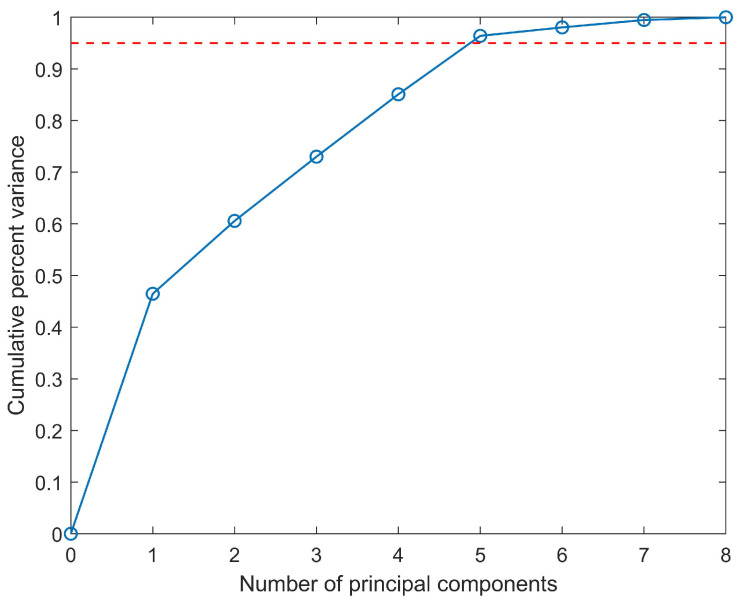
Cumulative Percent Variance of various principal components. The blue line represents the Cumulative Percent Variance. The red dotted line represents 95% explained variance.

The first step is offline modeling and extracting the feature in historical concentration data through PCA. Figure 6 shows the Cumulative Percent Variance in various principal components. When five principal components are selected, the Cumulative Percent Variance is greater than 95%. Therefore, the PCS contains the information of five principal components, while the remaining makes up the RS. Then, the monitoring indices, $Q_{UCL}$ and $T_{UCL}^{2}$, are calculated through Equations (16), (17), (26) and (27). When α=0.95, the results $T_{UCL}^{2}=10.8416$ and $Q_{UCL}=0.8454$ are obtained.

#### 4.2.2. APE Detection

**Figure 7:**
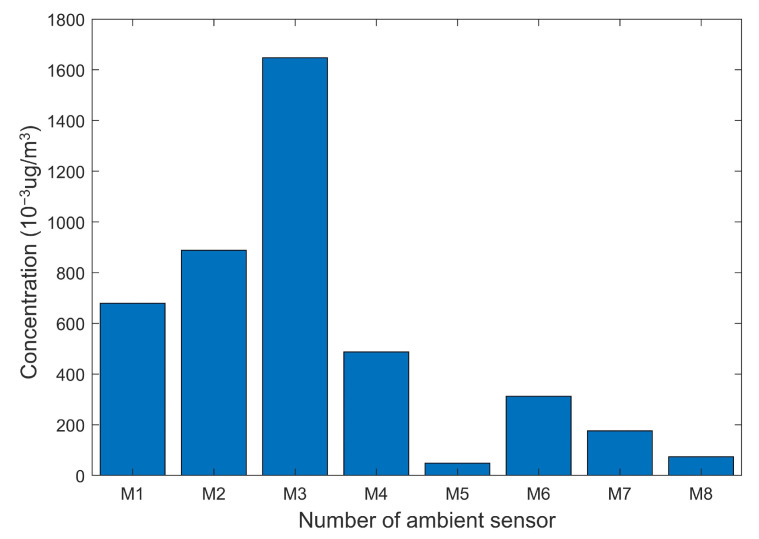
The concentration of the 300th test sample.

For the real-time APE detection, the 300th test sample caused by APE 6 (excess emission from Plant 6) is taken as an example. The concentration of this sample is shown in Figure 7. The real-time monitoring indices Q=0.0453 and $T^{2}=24.8027>T_{UCL}^{2}$, which meets the criteria proposed in Equation (29). Therefore, it can be determined that an APE caused by excessive emission is occurring.

Table 4 shows the APE detection rates for all six APEs. The results indicate that most APEs can be detected with almost 100% accuracy. Only APE 6 has a relatively low detection rate, but it is also greater than 90%. This proves that good performance is achieved by using the proposed method.

**Figure 8:**
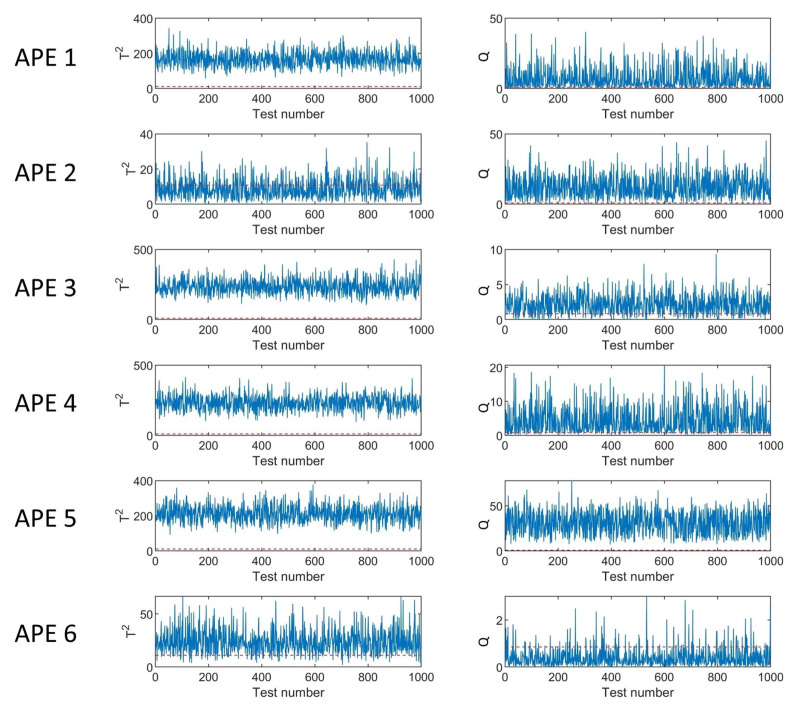
Monitoring charts in different kinds of APE. The blue line denotes $T^{2}$ or *Q* in various tests. The red dotted line represents the threshold of $T^{2}$ or *Q*.

The monitoring charts for each APE are shown in Figure 8. The solid curves represent the indices, while the dashed lines represent the upper control limits at a 95% confidence level based on KDE. It can be seen that the $T^{2}$ index is a more effective monitoring index for APE 1, 3, 4, 5, and 6, while *Q* index is a better choice for APE 2.

**Figure 9:**
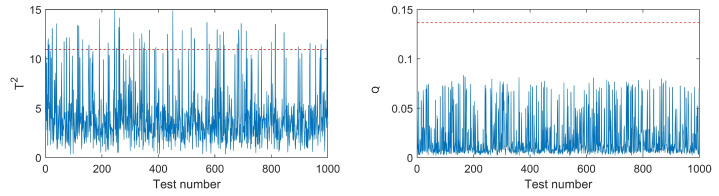
Monitoring charts under normal emission rates. The blue line denotes $T^{2}$ or *Q* in various tests. The red dotted line represents the threshold of $T^{2}$ or *Q*.

Figure 9 illustrates the monitoring charts under normal emission rates. It can be observed that the *Q* index is lower than the threshold at all time points, while the $T^{2}$ index exceeds the limit at several time points. The false alarming rate is 5.4%, which is maintained at a low level.

## 5. Real-World Application

This section demonstrates a practical trial in the CIP utilizing the proposed method based on real measurements. Unlike the simulation experiment, the whole park with 30 micro-stations is taken into account. Data of TVOC concentration and meteorological conditions are provided by the micro-stations at a 10 min time interval. This trial aims to detect the APEs caused by excessive TVOC emissions from 3 December 2020∼12 December 2020 using historical and real-time measurements.

### 5.1. Data

**Figure 10:**
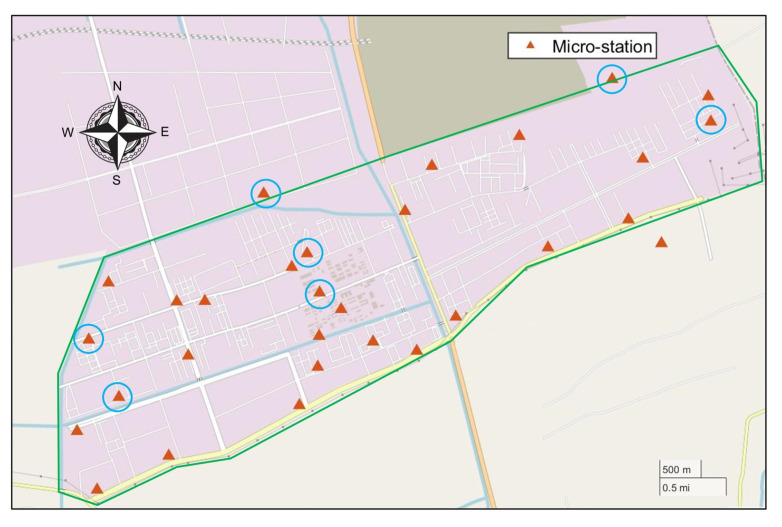
Map of the CIP. The green line represents the boundary of the CIP. Micro-stations circled in blue are stations with many missing data.

Due to the instability of gas sensors, the concentration readings from six micro-stations (which has been circled by blue circle in Figure 10) are significantly incomplete. Hence, the TVOC concentration measurements from the rest of 24 micro-stations are analyzed in this study.

The meteorological data (mainly including the wind direction and wind speed) used in the method are the average of the weather data collected from each micro-station. It is noted that when calculating the averages, special care should be given to wind direction, which cannot be calculated as a scalar. The wind is a vector quantity with a direction (wind direction θ) and a magnitude (wind speed *v*). There are two wind components, the east–west and north–south components. Therefore, wind direction and speed should be converted into these two components firstly before averaging. The specific method follows [48].

### 5.2. Analysis and Results

#### 5.2.1. Model Training and Updating

Potential APEs caused by excessive TVOC emissions from 3 December 2020∼12 December 2020 are detected following the flowchart shown in Figure 3. There are a few details in the process of modeling that should be highlighted. Firstly, it is necessary to classify the data according to wind direction before the model training. Various models and thresholds are established for different categories of data. In simulation experiments, every range of ten degrees is treated as a class. However, in practical applications, the number of data in each category may not be enough if this range is too small used for classification. Meanwhile, the wind directions in the studied period are concentrated at $100^{\circ }∼280^{\circ }$. Therefore, the range of wind directions in each type are $100^{\circ }∼130^{\circ }$, $130^{\circ }∼160^{\circ }$, $160^{\circ }∼190^{\circ }$, $190^{\circ }∼220^{\circ }$, $220^{\circ }∼250^{\circ }$, $250^{\circ }∼280^{\circ }$, and $280^{\circ }∼100^{\circ }$, respectively.

**Figure 11:**
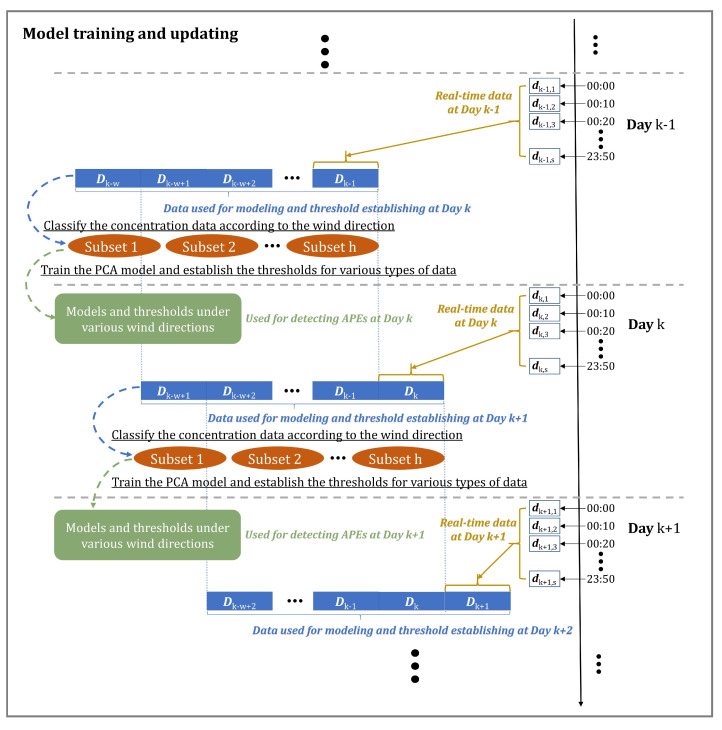
The process of model training and updating in the real experiment. $D_{i}$ is the data on *i*-th Day. *w* is the time range of the historical data. $d_{i,j}$ is the data at *j*-th time point on *i*-th Day, for j=1,2,3,⋯,s, where *s* is the total number of monitoring time points in a day. *h* is the number of the subsets classified by the wind direction.

In addition, the historical data used for training the models and establishing the thresholds should be updated in practice on a regular basis, causing the models and thresholds to change. Figure 11 illustrates the modeling process in the real experiment. It can be seen that the process of updating occurs only between 23:50 on the previous day and 0:00 on the current day, and the retrained models and thresholds are utilized to detect the APEs on the current day. Furthermore, the date range of selected historical data is fixed in this trial. If the range is too short, the historical data are not sufficient to reflect the statistical rules of pollution in recent periods. If the range is too long, the daily emission levels of the plants may change greatly due to the change in the production plan and products, which may have a great impact on the monitoring measurements. Therefore, the time range of historical data is empirically selected at 60 days (the value *w* shown in Figure 11 is 60, hence s=6×24×60=8640). For example, the models and thresholds used to detect APEs in 3 December 2020 are trained through 8640 groups of data from 4 October 2020 00:00∼2 December 2020 23:50.

#### 5.2.2. On-Line Monitoring

Take 1:50 on 4 December 2020 as an example to illustrate the online monitoring process. Before 00:00 on this day, 8640 groups of historical data from 5 October 2020 00:00∼3 December 2020 23:50 were selected. After classification based on the wind direction, the features of each type were extracted through PCA, and the models and thresholds of each type were obtained. In real-time monitoring, the monitoring indices, $T^{2}$ and *Q*, were calculated to determine whether the air pollution episodes had occurred. At 10:50 on 8 December 2020, the wind direction was $208.0^{\circ }$, which belonged to the type of $190^{\circ }∼220^{\circ }$. For this type, $T_{UCL}^{2}=3.8920$ and $Q_{UCL}=1.8977$ with 95% confidence. An APE was detected at this time because the real-time index $Q=2.1840>Q_{UCL}$.

**Figure 12:**
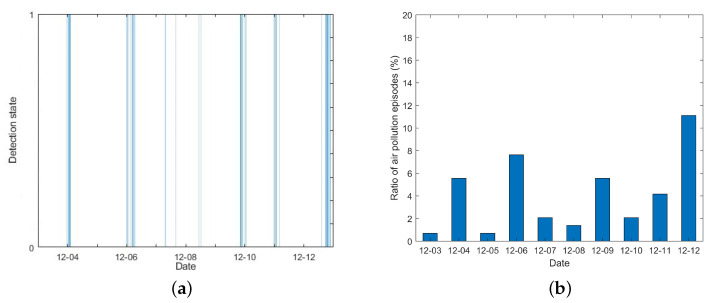
Air pollution episode detection results. Detection state. (**a**) If the detection rate is 1, an APE is detected. (**b**) Ratio of air pollution episodes detected per day.

Figure 12 demonstrates the APE detection results from 3 December 2020 00:00 to 12 December 2020 23:50. Over a 10-day period, 64 APEs were detected in 1440 time samples, and the ratio of APEs is 4.1%. The detection state is shown in Figure 12a, and Figure 12b depicts the ratio of APEs detected per day. It can be seen that the abnormal situation mainly occurred in 4 December 2020, 6 December 2020, and 12 December 2020. This means that some enterprises in the CIP emitted excessively during this time period, which may be due to increased production activities or unreasonable emissions in pursuit of economic benefits.

## 6. Conclusions and Outlooks

### 6.1. Conclusions

This paper proposes a new framework using multivariate statistical analysis to detect air pollution episodes in CIPs. This method can help park managers to identify APEs due to excessive emissions in the CIPs timely and effectively. In this paper, the PCA approach is studied in particular. The monitoring indices $T^{2}$ and *Q* are utilized in APE detection. The limits of these indices are based on the historical data, which can reflect the air pollution status better and make the APE detection more efficient compared with fixed environmental standards. At the same time, the concept of APE detectability is also introduced, and the condition of APE detectability is derived to explain when the APEs can be detectable.

A simulation experiment is presented to detect the abnormal situation caused by excess emissions in a part of a CIP in Zhejiang province, China. A large number of historical monitoring data are used to train the data-driven models and create the limits of monitoring indices, which provide the basis of pollution detection. The result shows that the approach can detect excess emissions effectively. Furthermore, the real TVOC data measured from the micro-stations in the CIP are utilized for APE detection, and 64 APEs caused by excessive TVOC emissions are detected in 1440 time points.

### 6.2. Limitations and Outlooks

1. The PCA-based monitoring approach is a steady-state monitoring method. In fact, the diffusion of air contaminants is a dynamic process. On the one hand, it will take a certain amount of time for the pollutants to be monitored. On the other hand, changes in wind speed and wind direction cannot be reflected in the monitored concentration quickly. Therefore, the dynamic characteristics of the data should be considered and some dynamic monitoring methods such as dynamic PCA (DPCA) and canonical variable analysis (CVA) can be utilized in future studies.
2. This method is based on the linear source–receptor relationship. Actually, there are some factors, such as the chemical reaction between pollutants and the decomposition of the contaminants, that break the linear relationship. Accordingly, the measurements and the source emission rates are not strictly linear, which does not meet the applicable conditions of the approach. In addition, the inverse relationship between concentration and wind speed does not hold under the condition of low wind and still wind. In future research, more complex and dynamic models will be utilized to analyze the source–receptor relationship. Some non-linear methods, such as kernel PCA (KPCA) and artificial neural network (ANN), can be used.
3. In this paper, a theoretical definition of APE detectability is proposed to give a condition to determine whether the APEs in CIPs can be detectable. However, APE detectability is not discussed in depth in this paper. In fact, it can serve as a theoretical basis to evaluate the performance of existing AQMNs and design a new AQMN, which can be discussed further in the future. Furthermore, the APE detection methods when the APE detectability condition is not satisfied can also be studied in future research.

## Figures and Tables

**Table 1:** Localization parameter settings of AERMOD.

Parameter	Value
Albedo	0.2075
Bowen Ratio	1.625
Surface Roughness	0.3

**Table 2:** Setting of source emission rates.

Parameter	Explanation	Value	Unit
$q_{A}$	emission rate of source *A*	$q_{A}∼N\left(359,14.7\right)$	mg/s
$q_{B}$	emission rate of source *B*	$q_{B}∼N\left(236,10.3\right)$	mg/s
$q_{C}$	emission rate of source *C*	$q_{C}∼N\left(218,7.2\right)$	mg/s
$q_{D}$	emission rate of source *D*	$q_{D}∼N\left(315,5.9\right)$	mg/s
$q_{E}$	emission rate of source *E*	$q_{E}∼N\left(365,5.1\right)$	mg/s
$q_{F}$	emission rate of source *F*	$q_{F}∼N\left(276,10.9\right)$	mg/s

**Table 3:** The description of APE.

APE	Description
1	Excess emission from Plant A
2	Excess emission from Plant B
3	Excess emission from Plant C
4	Excess emission from Plant D
5	Excess emission from Plant E
6	Excess emission from Plant F

**Table 4:** Test accuracy of APE detection.

APE	1	2	3	4	5	6
**Test accuracy** (%)	100	99.8	100	100	100	91.1

## Data Availability

Not applicable.

## Abbreviations

The following abbreviations are used in this manuscript:

APE	Air pollution episode
CIP	Chemical industry park
AQMN	Air quality monitoring network
STE	Source term estimation
UAV	Unmanned aerial vehicle
VOC	Volatile organic compound
MSA	Multivariate statistical analysis
PCA	Principal component analysis
PCS	Principal component subspace
RS	Residual subspace
CPV	Cumulative Percent Variance
PDF	Probability density function
KDE	Kernel density distribution
